
JOURNAL INFORMATION
==============================
NLM Title Abbreviation: ScientificWorldJournal
Journal ID: ScientificWorldJournal
NLM Journal ID: 101131163
Journal ID: TSWJ

The Scientific World Journal

ISSN: 2356-6140
EISSN: 1537-744X
Publisher: Wiley

ARTICLE INFORMATION
==============================
PMCID: PMC5763926
PMID: 20526528
DOI: 10.1100/tsw.2010.92
Article ID: 930717
Article version: 1
Subjects: Clinical Images

Anal Carcinoma in an HIV-Infected Woman

Sendagorta Elena 1 *
Herranz Pedro 1
Guadalajara Héctor 2
Zamora Francisco 3
Gonzalez Juan 3
1 Department of Dermatology, University Hospital La Paz, Madrid, Spain
2 Department of Surgery, University Hospital La Paz, Madrid, Spain
3 HIV Unit, Internal Medicine Department, University Hospital La Paz, Madrid, Spain
*Elena Sendagorta: elenasendagorta@hotmail.comAcademic Editor: Daniel Batlle

Publication date: 2010
Electronic publication date: 2010 Jun 1
Volume: 10
First page: 986
Last page: 987
Received 2010 Apr 11; Revised 2010 Apr 26; Accepted 2010 Apr 28
Copyright: Copyright © 2010 Elena Sendagorta et al.
Copyright year: 2010
License: This is an open access article distributed under the Creative Commons Attribution License, which permits unrestricted use, distribution, and reproduction in any medium, provided the original work is properly cited.
License URL: https://creativecommons.org/licenses/by/3.0/

Keywords: anal carcinoma, anal intraepithelial neoplasia, HIV infection, HPV infection

==============================
Clinical Image
*TheScientificWorldJOURNAL* (2010) 10, 986–987
ISSN 1537-744X; DOI 10.1100/tsw.2010.92

TheScientificWorldJOURNAL
www.thescientificworld.com

### Anal Carcinoma in an HIV-Infected Woman

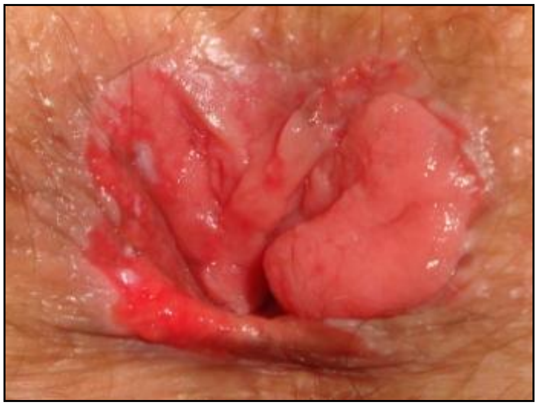

Elena Sendagorta[1,*], Pedro Herranz[1], Héctor Guadalajara[2], Francisco X. Zamora[3], and Juan Gonzalez[3]
*[1]Department of Dermatology, [2]Department of Surgery, and [3]HIV Unit, Internal Medicine department, University Hospital La Paz, Madrid*

**E-mail**: elenasendagorta@hotmail.com

*Received April 11, 2010; Revised April 26, 2010; Accepted April 28, 2010; Published June 1, 2010*

**KEYWORDS:** anal carcinoma, anal intraepithelial neoplasia, HIV infection, HPV infection

A 42-year-old woman presented with an asymptomatic lesion of nearly 6-months duration on the right surface of the anal canal mucosa. She had an 18-year history of HIV infection on triple antiretroviral therapy. Her CD4 count was 420 cells/$mm^3$ and viral load was undetectable. As a remarkable antecedent, 6 years ago, she underwent a conization because of a high-grade intraepithelial cervical neoplasia (CIN) and a partial vulvectomy because of an intraepithelial vulval neoplasia (VIN), both related to an HPV 16 infection.

Physical examination showed a 3- × 2-cm tumor proximal to the right side of the anal verge. Microscopic evaluation of a lesional biopsy led to the diagnosis of squamous cell carcinoma of the anal canal. HPV 16 was detected again by PCR on the tissue sample.

The incidence of anal cancer is increasing among several important subgroups of the population, in particular among people with HIV. In a recent study, the incidence rate of anal cancer in an HIV-infected cohort reached 128/100,000 person-years during 2006–2008[1].The incidence of anal cancer is greatest in men who have sex with men (MSM) with reported rates of 70–224/100,000 population[2].

Among women with HIV, the incidence of invasive anal cancer is 7–28 times higher than in the general population, most of them being squamous cell carcinomas[3]. Women with CIN or VIN are more

*Corresponding author.
©2010 with author.
Published by TheScientificWorld; www.thescientificworld.com likely to have anal high-risk HPV infection and anal cancer[4]. As shown in this case, anogenital HPV disease is probably multicentric, and cervical HPV infection may serve as a reservoir for anal HPV infection and vice versa[4]. In fact, the prevalence of HPV infection–related disease in HIV-infected women is higher in the anus than in the cervix[5]. Thus, careful follow-up and search for anal disease is mandatory in HIV-infected women, and, above all, in those with an antecedent of genital HPV infection.

### REFERENCES

1. Crum-Cianflone, N.F., Hullsiek, K.H., Marconi, V.C., Ganesan, A., Weintrob, A., Barthel, R.V., Agan, B.K.; Infectious Disease Clinical Research Program HIV Working Group (2010) Anal cancers among HIV-infected persons: HAART is not slowing rising incidence. *AIDS* **24,** 535–543.
2. Kreuter, A., Brockmeyer, N.H., Altmeyer, P., Wieland, U.; German Competence Network HIV/AIDS (2008) Anal intraepithelial neoplasia in HIV infection. *J. Dtsch. Dermatol. Ges.* **6,** 925–934.
3. Frisch, M., Biggar, R.J., and Goedert, J.J. (2000) Human papillomavirus-associated cancers in patients with human immunodeficiency virus infection and acquired immunodeficiency syndrome. *J. Natl. Cancer Inst.* **92,** 1500–1510.
4. Goodman, M.T., Shvetsov, Y.B., McDuffie, K., Wilkens, L.R., Zhu, X., Thompson, P.J., Ning, L., Killeen, J., Kamemoto, L., and Hernandez, B.Y. (2010) Sequential acquisition of human papillomavirus (HPV) infection of the anus and cervix: The Hawaii HPV Cohort Study. *J. Infect. Dis.* **201,** 1331–1339.
5. Palefsky, J.M., Holly, E.A., Ralston, M.L., Da Costa, M., and Greenblatt, R.M. (2001) Prevalence and risk factors for anal human papillomavirus infection in human immunodeficiency virus (HIV)-positive and high-risk HIV-negative women. *J. Infect. Dis*. **183,** 383–391.

**This article should be cited as follows:**

Sendagorta, E., Herranz, P., Guadalajara, H., Zamora, F.X., and Gonzalez, J. (2010) Anal carcinoma in an HIV-infected woman. *TheScientificWorldJOURNAL* **10**, 986–987. DOI 10.1100/tsw.2010.92.
